# Neural dynamics of feedforward and feedback processing in figure-ground segregation

**DOI:** 10.3389/fpsyg.2014.00972

**Published:** 2014-09-10

**Authors:** Oliver W. Layton, Ennio Mingolla, Arash Yazdanbakhsh

**Affiliations:** ^1^The Perception and Action Lab, Department of Cognitive Science, Rensselaer Polytechnic InstituteTroy, NY, USA; ^2^Vision Lab, Center for Computational Neuroscience and Neural Technology, Boston UniversityBoston, MA, USA; ^3^Computational Vision Laboratory, Department of Speech-Language Pathology and Audiology, Northeastern UniversityBoston, MA, USA

**Keywords:** V4, figure-ground segregation, medial axis transform, ventral stream, feedforward, feedback

## Abstract

Determining whether a region belongs to the interior or exterior of a shape (figure-ground segregation) is a core competency of the primate brain, yet the underlying mechanisms are not well understood. Many models assume that figure-ground segregation occurs by assembling progressively more complex representations through feedforward connections, with feedback playing only a modulatory role. We present a dynamical model of figure-ground segregation in the primate ventral stream wherein feedback plays a crucial role in disambiguating a figure's interior and exterior. We introduce a processing strategy whereby jitter in RF center locations and variation in RF sizes is exploited to enhance and suppress neural activity inside and outside of figures, respectively. Feedforward projections emanate from units that model cells in V4 known to respond to the curvature of boundary contours (*curved contour cells*), and feedback projections from units predicted to exist in IT that strategically group neurons with different RF sizes and RF center locations (*teardrop cells*). Neurons (*convex cells*) that preferentially respond when centered on a figure dynamically balance feedforward (bottom-up) information and feedback from higher visual areas. The activation is enhanced when an interior portion of a figure is in the RF via feedback from units that detect closure in the boundary contours of a figure. Our model produces maximal activity along the medial axis of well-known figures with and without concavities, and inside algorithmically generated shapes. Our results suggest that the dynamic balancing of feedforward signals with the specific feedback mechanisms proposed by the model is crucial for figure-ground segregation.

## Introduction

Figure-ground segregation refers to the process by which the visual system parses the complex array of luminance that appears on the retina into perceptually grouped foreground objects (figures) and backgrounds (ground). To distinguish between figures and their background, the visual system must perform two complementary processes—detecting defining borders and integrating parts into wholes. How the visual system represents visual figures with respect to these two processes, and the underlying mechanisms, are largely unknown. Emerging neurophysiological and psychophysical evidence suggests that the visual system may rely on multiple parallel “solutions” to segment the visual scene into figures and backgrounds.

One solution likely involves the border-ownership assignment of local edge representations. Figures necessarily share a visual border of an adjacent background region, and border-ownership refers to the association of the border with the figure rather than the ground. Populations of edge-sensitive neurons in primate visual areas V1, V2, and V4 have been shown to exhibit sensitivity to border-ownership: neurons respond with a higher firing rate when the figure to which the edge in the receptive field (RF) is attached appears on the preferred side (Zhou et al., 2000). If the figure is on the other side of the edge, then the firing rate of the neuron will decrease and another neuron will exhibit enhanced activity. Neural models have suggested that border-ownership selectivity may arise through feedback from neurons with larger RFs in higher visual areas (Kelly and Grossberg, 2000; Craft et al., 2007; Jehee et al., 2007; Layton et al., 2012), through feedforward processing alone (Supèr et al., 2010), or through horizontal connections within V2 (Zhaoping, 2005). Border-ownership signals require no more than 25 ms from the presentation of the figure to emerge (Zhou et al., 2000), which constrains the set of possible mechanisms. In early visual areas, feedback connections have the fastest conduction velocities (~3.8 m/s) that are considerably faster than those of horizontal connections (~0.3 m/s; Girard et al., 2001). Feedback connections are likely involved in border-ownership because they span large cortical areas with minimal delay, unlike horizontal connections.

**Figure 1:**
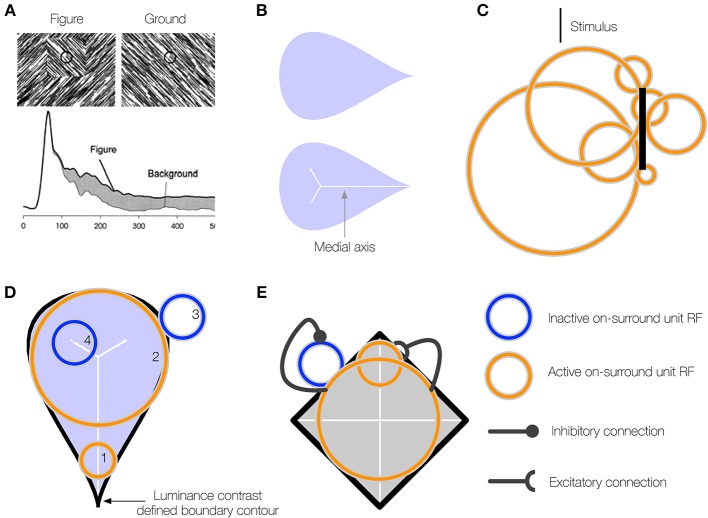
**(A)** A neuron in primate V1 demonstrates an increased firing rate (*interior enhancement*) when the RF is centered on the interior of a figure compared to a background. Left: A square figure defined by the convergence of lines with two different orientations. The black circle at the center of the square depicts the classical RF of the V1 neuron. Right: A homogeneous background. Bottom: The response of the V1 neuron is greater when the RF is centered on the square figure than the homogenous background. Interior enhancement in the neuron's response occurs, despite the fact that the classical RF is positioned far from the orientation-defined boundary of the square and the visual pattern in the RF is the same in the figure and background displays. Figure reproduced from Roelfsema et al. (2002). **(B)** A teardrop figure (top) and its medial axis superimposed (bottom). Medial axes are computed using the built-in function in Mathematica and thickened for clear visibility. **(C)** A minimal bar stimulus activates a number of neurons in cortex, with displaced RF centers and variable RF sizes (jitter). **(D)** A population of neurons with jittered RF positions and sizes can detect the medial axis of a figure. Units 1–2 respond strongly when their on-surround, annulus-shaped RFs are centered on certain points along the medial axis of the teardrop figure. The response is driven by contact between the annulus and the boundary contours, defined by luminance contrast (black). The response is weak when the RF is not centered along the medial axis (3) or the RF size of a unit centered along the medial axis is too small (4) or large compared to the boundary. **(E)** On-surround units may falsely respond outside of a figure due to the presence of a boundary contour in the RF (blue). Feedback from units with large RFs, which provide a measure of the closure of the figure boundary, can enhance the activity of units whose RFs are centered on the interior of a figure (orange) and suppress due to the background (blue).

Another solution likely involves an enhancement of neural activity to the interior surface of the figure compared to the exterior (*interior enhancement*). When Lamme and colleagues centered the interior of a texture-defined square within the RF of neurons in early visual areas of monkey, the neurons exhibited an enhanced firing rate compared to when the monkeys were presented a uniform texture (Figure 1A; Lamme, 1995; Zipser et al., 1996). The interior enhancement effect persists when the edges of the square are 8–10° and the modulation occurs after an 80–100 ms latency from the onset of the stimulus, which suggests feedback from neurons with larger RFs may be involved. A temporal analysis indicates that neural activity relating to the edges of the figure emerge first, following a short latency, then interior enhancement occurs in the “late component” of the response (Lamme et al., 1999; Lamme and Roelfsema, 2000). A neuron that shows interior enhancement continues to fire at an elevated rate when the RF is centered at different positions within the texture-defined figure, and the firing rate drops precipitously when the RF is centered on the background (Lee et al., 1998; Friedman et al., 2003). Neurons in V2 demonstrate a greater degree of interior enhancement compared to those in V1 (50% vs. 30%; Marcus and Van Essen, 2002), and the magnitude of interior enhancement response is greatest in V4 (50% greater than in V1; Poort et al., 2012).

Our understanding of the mechanisms underlying interior enhancement of figures is poor. Given that interior enhancement has only been demonstrated in primary visual cortex, occurs with figures many times larger than the classical RF, and is associated with the late component of the neural response, we wondered if higher visual areas may underlie the effect. That is, we hypothesize that interior enhancement first occurs in higher visual areas and propagates via feedback to early visual areas. Neurons in higher visual areas have larger RF sizes and are ideally suited to determine whether a region belongs to the interior or exterior of a figure. Recurrent connections and multiple feedback loops with early visual areas may explain the late onset latency of interior enhancement.

If higher visual areas mediate the effect, what are neurons with limited RF sizes in early visual cortex that demonstrate interior enhancement signaling about the interior of a figure? We propose that interior enhancement is a means to code the figure with respect to its medial axis (Burbeck and Pizer, 1995; Kovács et al., 1998; Pizer et al., 1998). The medial axis (“skeleton”) of a figure defines the set of points along the interior that run equidistant to points along the boundaries (Figure 1B). It is a compact representation of the shape. The “late component” response of neurons in the primate ventral stream that is characteristic of interior enhancement (Lee et al., 1998) has also been associated with a response to the medial axis of shapes, particularly in inferotemporal cortex (IT; Hung et al., 2012). In humans, fMRI BOLD signals related to the medial axis first emerge in areas V3 and beyond in the ventral stream (Lescroart and Biederman, 2013), which indicates that higher visual areas are important for detecting the medial axis. Medial selectivity in higher visual areas and the late onset of the modulation in early cortical areas suggest that interior enhancement is not a solely feedforward phenomenon. Psychophysical evidence demonstrates that humans exhibit a heightened sensitivity to the medial axis of shapes (Wang and Burbeck, 1998). Julesz and colleagues presented humans with an array of randomly oriented Gabor patches, except for those that collectively composed the boundary of shapes, such as ellipses, cardioids, and triangles (Kovács et al., 1998). Subjects performed a differential contrast detection task of a Gabor pattern that lay some distance on the interior of the shape boundary, and threshold performance was mapped out. The contrast sensitivity of subjects was greatest along the medial axis and the spatial profile of thresholds matched the medial axis representations at different spatial scales. These results indicate that the visual system is particularly sensitive to a figure's medial axis. The medial axis plays an important role in the Core theory of Pizer and colleagues that posits that the visual system represents a figure with respect to its boundary, middle, and width at multiple spatial scales (Pizer et al., 1998). As explained below, the central innovation of the present work is to show that medial representations at multiple spatial scales hold a key to figure-ground segregation, when combined with RF jitter and cooperative-competitive dynamics across neurons in multiple areas of the primate visual system.

If neurons that exhibit interior enhancement code the medial axis of a figure, how do these neurons integrate information about the boundary, given that the classical RF size of a single neuron is fixed and the distance between the medial axis and the boundary may vary? Not many models address the variability in RF sizes in areas of cortex. Contrary to the classical view that a minimal stimulus, such as a small bar, activates neurons with small non-overlapping RFs early in cortex, the neurons that respond to the stimulus occupy a small patch of cortex known as the cortical “point spread” (Das and Gilbert, 1995). Neurons within the “point spread” tend to be spatially close in cortex, but possess a diverse range of RF centers and sizes (Figure 1C; Gilbert et al., 1996). We use the term *jitter* to refer to the displacement of RF centers and variation in RF sizes among nearby neurons in cortex. Within and across visual areas along the ventral stream, RF size and jitter grows proportionately with eccentricity (Gattass et al., 1981, 1988; Bakin et al., 2000). Our model proposes that one of the functions of the naturally occurring jitter in the visual system is to locally “probe” for the medial axis of figures. The activation of some, but not all, neurons with displaced RF centers and sizes within a small patch of cortex provides detailed information about where the medial axis is likely positioned and its spatial extent (Figure 1D). Neurons with a single RF size may not be able to signal the presence of the medial axis of a figure in general.

Our model solves a crucial problem through feedback and the recruitment of neurons with multiple RF sizes that compute a scale-sensitive estimate of the medial axis of a figure. Although a pair of equidistant contours may locally appear within the RF, the contours may not belong to a figure (Figure 1E). The contours may be incomplete fragments or lie outside of a perceived figure, in which case neurons that demonstrate interior enhancement do not fire (Lee et al., 1998). The visual system appears particularly sensitive to the Gestalt *closure* of a figure's boundary contours, whether they are continuous or fragmented (Elder and Zucker, 1993; Kovács and Julesz, 1993; Gerhardstein et al., 2004; Mathes and Fahle, 2007). We propose that neurons in IT cortex that respond to configurations of contours provide a measure of a figure's closure (Brincat and Connor, 2004, 2006). In our model, signals that emerge from units that collect evidence about a figure's closure send feedback to suppress the activity of units that codes the medial axis when their RFs are centered outside of figures (see blue unit, Figure 1E).

Here we introduce a neural model, called *the teardrop model*, to investigate the hypothesis that interior enhancement occurs in higher visual areas and underlies the effect observed in the primary visual cortex. The model is a multi-level network, consisting of cooperative/competitive interactions at each stage. In the context of figure-ground segregation, models that implement cooperative and competitive dynamics identify a global solution in the large space of possible interpretations (Edelman, 1987; Grossberg, 1994). Units in the teardrop model capitalize on jitter to reinforce the representation of the global figure and suppress other interpretations. We use areas (e.g., V1, V4, etc.) when referring to model layers, analogous to the areas in primate cortex that we believe carry out similar functions and dynamics. To focus on fundamental figure-ground mechanisms, the retina, LGN, and V1 are simplified and lumped together in a preliminary model stage that generates an edge map of figures in the visual display, as is thought to occur in early visual areas. The model has stages corresponding to areas V4, posterior IT (PIT), and anterior IT (AIT). Our model is consistent with physiological evidence that IT sends extensive feedback projections to V4 (Gattass et al., 1988; Piñon et al., 1998). As will be explained below, neurons analogous to those in IT may combine figure representations at multiple spatial scales and propagate information back to neurons that estimate the position of the medial axis.

**Figure 2:**
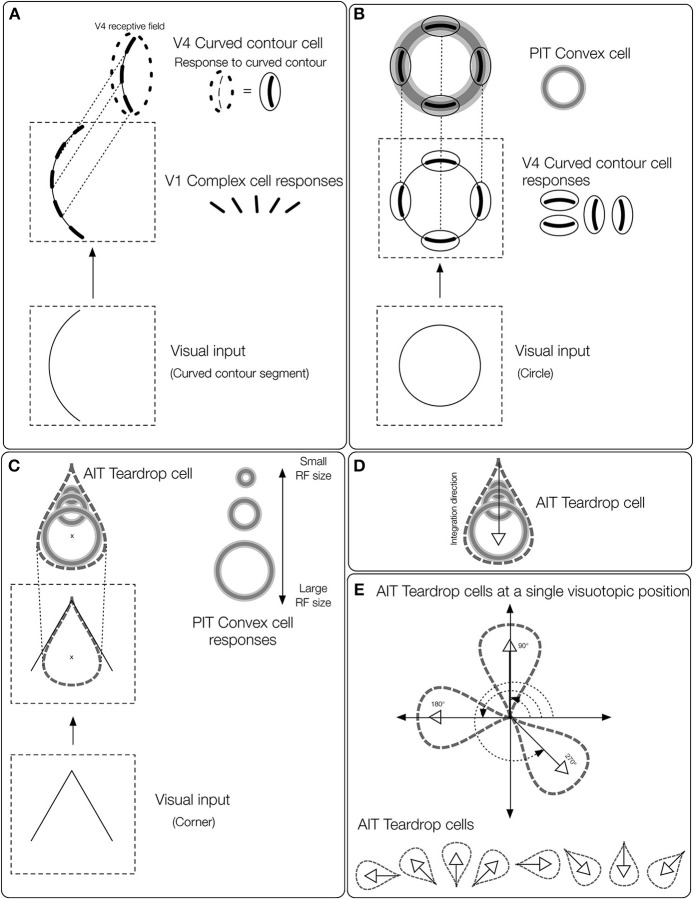
**Overview of the teardrop model stages**. Network layers are labeled (e.g., V4, PIT, etc.) according to where the computations are proposed to take place in the primate visual system. The input to the model is a preprocessed edge map of the visual display, similar to the output of V1 complex cells. **(A)** The first model stage contains cells selective to curved contours (*curved contour cells*). When a curved segment enters the RF (bottom panel), curved contour cells group the piecewise linear spatial pattern of complex cell outputs (middle panel) to approximate a curved segment (top panel). The dashed ellipse signifies the curved contour cell RF, which hereafter is represented by a curved segment embedded inside a solid ellipse. **(B)** *Convex cells* in model PIT receive input from curved contour cells in an on-surround/annular spatial arrangement. Convex cells respond optimally to circles (bottom panel), because curved contour cell responses to the circular boundary contours perfectly coincide with the annular receptive field of the convex cell (top panel). Convex cells respond to points along the medial axis of a figure because the units receive input from equidistant curved contour signals about the boundary. **(C)** Model AIT cells are called *teardrop cells* and respond to an ordered (by scale) collection of convex cell outputs along a medial axis segment. The “x” marks the visuotopic position of the teardrop cell RF. Teardrop cells that share the same RF position also receive input from the convex cell whose RF center is marked by the “x.” **(D)** The shown teardrop cell groups convex cells with RF sizes increasing with distance from the base of the arrow and estimates the medial axis of the corner input. Teardrop cells are hereafter depicted by the teardrop outline. **(E)** In our simulations, teardrop cells whose RFs are positioned at a single visuotopic location have one of eight integration directions, indicated by the white outlined arrows.

The mechanisms in the teardrop model bring together aspects of the visual system to support figure-ground segregation in a method not described before. Our model consists of three main propositions. (1) Neurons that show an enhanced response to the interior of a figure signal the figure's medial axis (Figure 1B). (2) The visual system detects the figure's medial axis by recruiting neurons with jittered RF sizes and positions (Figure 1D). (3) Feedback from higher visual areas is necessary to constrain neural responses to the interior of figures (Figure 1E). Model convex cells (model PIT) exhibit enhanced responses to the interior of a figure after the following sequence of operations:

- Start with a V1 complex cell-like edge representation of the boundary contours.
- Detect curved contours by grouping the edge segment output of the complex cells (Figure 2A).
- Estimate points along the medial axis of the figure using convex cells (Figure 2B).
- Detect closure in boundary contour segments by integrating points along the medial axis via teardrop cells (Figures 2C–E). Teardrop cells are an ordered (by scale) collection of convex cell outputs along a medial axis segment.
- Suppress activity in convex cells to concave regions of the figure (**Figure 4**).
- Suppress activity in convex cells on the exterior of the figure using teardrop cells (**Figure 5**).

To our knowledge, the model created by Roelfsema and colleagues is the only existing investigation of the mechanisms underlying interior enhancement of a figure on a background. The model, however, is restricted to simple texture-defined squares and does not consider more complex shapes and visual scenes (Roelfsema et al., 2002). Our model is capable of performing figure-ground segregation in scenes with any number of figures, whose boundaries form simple closed curves or incomplete fragments thereof. We test our model on images of natural scenes and parametrically generated shapes with varying numbers and degrees of concavities. Our model also addresses response enhancement to a figure's interior in line-drawing or representations of figures whose boundary contours are not continuous. We do not address perceptual grouping that occurs behind occlusion. Several properties emerge through the dynamics of our model that are consistent with physiological data, such as the size-invariant response properties of IT neurons (Appendix 1 in Supplementary Material; Ito et al., 1995; Logothetis et al., 1995).

## Materials and methods

**Figure 3:**
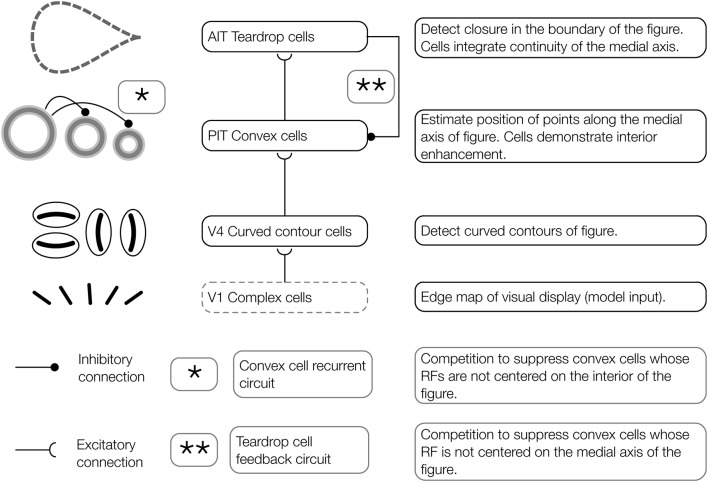
**Architecture of the proposed model of figure-ground segregation**. Convex cells in the model demonstrate interior enhancement when their RFs are centered along the medial axis of a figure. Preprocessed edge maps of each visual display serve as input to the model. The input contains the edges of potential figures and roughly corresponds to the output of complex cells in primate V1. In the first model layer, curved contour cells detect the curvature of edges in the visual display. Curved contour cells project to convex cells in the second model layer, which possess on-surround, annulus-shaped RFs. Convex cells respond when the boundary contours of a figure enter the parameter of the circle depicting the RF. These units are ideally suited for detecting points along the medial axis of a figure. A central claim of the model is that the visual system exploits jitter in the RF size and position to perform figure-ground segregation. Teardrop cells group signals from convex cells with different RF sizes and positions to detect closure in the boundary of a figure and the medial axis. Feedback from teardrop cells (pathway ^**^, teardrop cell feedback circuit) enhances the activity of convex cells centered along the medial axis of a figure (interior enhancement), and suppresses activity elsewhere. In the convex cell recurrent circuit (pathway ^*^), convex cells with large RFs send recurrent feedback to convex cells with smaller RFs to suppress responses to regions outside of figures (concavities).

The aim of the present study is to have a better understanding of how interior enhancement occurs in the primate visual system. We use the model to test the hypothesis that dynamical feedforward and feedback interactions with higher visual areas in the ventral stream give rise to interior enhancement. Our model consists of three network layers that we believe correspond to primate visual areas V4, posterior inferotemporal cortex (PIT), and anterior inferotemporal cortex (AIT). We find these areas candidates for the computations carried out by the model based on evidence referenced below. Properties of model curved contour and convex cells are based on known physiology in corresponding areas, and those of teardrop cells are proposed. The proposed model is schematized in Figure 2 and the model stages are depicted in Figure 3.

### Model V4: curved contour cells

The inputs to the model are preprocessed edge maps, which approximate the output of complex cells in primary visual cortex (V1). We refer to the result of complex cells rather than simple cells because the edge maps are contrast polarity insensitive. The first layer of our model corresponds to area V4 in primate cortex (Figure 2A). We simulate the dynamics of cells sensitive to the curvature (*curved contour cells*). The behavior of model curved contour cells is similar to that of populations of V4 neurons, which, unlike those in V1 and V2, demonstrate far greater selectivity for curved contours (Pasupathy and Connor, 1999, 2001, 2002) and conjunctions of bars (Hegde and Van Essen, 2006; Yau et al., 2012) at multiple spatial scales (Mineault et al., 2013). Model curved contour cells respond optimally when a contour, such as a curved segment or corner, enters the RF that matches the unit's RF size and preferred curvature sensitivity (Figure 2A). At each visuotopic position, we simulate curved contour units tuned to eight arcs about a circle. We construct curved contour units with seven different RF sizes.

### Model PIT: convex cells

Curved contour cells in model V4 project to the second model layer, which corresponds to primate area PIT (Figure 2B). The purpose of model units in this network layer is to detect points along the medial axis or “skeleton” of figures. As shown in Figure 1D, units that integrate their curved contour inputs in an on-surround fashion, in the shape of an annulus, are ideally suited for detecting the medial axis because they receive bottom-up feedforward signals from the boundary contours when their RFs are centered on the figure. However, units with a single RF size are not sufficient for detecting the medial axis in general. Figure 1B shows that in the case of a teardrop shape, the distance changes between points along the medial axis and the boundary. Therefore, units with a single RF size are not sufficient for signaling the location of a figure's medial axis. A subset of units with different RF sizes can detect the medial axis, as indicated in Figure 1D by the active units. We call units that detect the medial axis *convex cells* (Figure 2B).

Convex cells simulate a number of properties from known neurophysiology and are consistent with findings from psychophysical experiments. Humans demonstrate a bias to judge symmetric, convex regions as figure, and asymmetric, concave regions as the background (Peterson and Salvagio, 2008; Kim and Feldman, 2009). Two dimensional shapes are more rapidly detected (Elder and Zucker, 1993) with higher accuracy (Kovács and Julesz, 1993; Mathes and Fahle, 2007) in humans, even at a young age (Gerhardstein et al., 2004), when the collection of boundary contours form a continuous closed curve, as opposed to when constituent contours possess different curvatures and orientations that do not align with the overall shape of the figure. These findings are consistent with the possibility that the visual system contains mechanisms that afford sensitivity to convexity and closure (Wagemans et al., 2012). Neurons in PIT appear to integrate multiple curved contour segments when they appear at particular orientations and positions within the RF (Brincat and Connor, 2004, 2006). For example, a neuron in PIT may optimally respond to a crescent shape because a number of curved segments that form the boundary contours appear together in appropriate positions in the RF (Brincat and Connor, 2004). The annulus has been shown to be an optimal stimulus for many neurons in intermediary areas of the ventral stream (Pollen et al., 2002; Hegde and Van Essen, 2006). An annular RF affords sensitivity to the figure-ground Gestalt properties of convexity and closure.

### Model AIT: teardrop cells

Units in the third model layer, model AIT, receive feedforward input from convex cells (Figure 2C). The purpose of units in the third network layer is to collect evidence about the presence of a continuous medial axis that spans the interior of a figure. While convex cells detect probable *points* along a figure's medial axis, more is needed to detect its full extent. Units in model AIT spatially integrate signals from convex cells. Recall that the collection of convex cells with a single RF size is in general insufficient for detecting the medial axis of a shape (Figure 1D). Therefore, units in model AIT integrate convex cells with different RF positions and sizes (Figure 2C). For example, the active units shown in Figure 1D collectively signal the medial axis of the teardrop shape.

To integrate signals from convex cells that have different RF sizes and positions, units in model AIT have RFs elongated in a particular spatial direction (*integration direction*). For example, the unit in model AIT that groups the set of convex cell units depicted in Figure 2D is elongated in the vertical direction, and therefore has a vertical integration direction. Hence, AIT units respond to the output of convex cells, ordered by scale along a common axis. In our simulations, we used eight integration directions at every location in the visual field (Figure 2E). The use of integration directions capitalizes on the jitter in RF size and position found in cortex. We found that units in model AIT that group feedforward signals from convex cells whose RF sizes linearly increase along the integration direction were sufficient for detecting the medial axis in the displays we consider. Therefore, we call units in model AIT *teardrop cells*.

We define the *position* of a teardrop cell's RF to coincide with the RF center of the largest convex cell that sends feedforward input. For example, the “x” marks the position of the teardrop cell depicted in Figure 2C. Teardrop cells with different integration directions at the same RF position share a common input from the largest convex cell that falls within the RF.

**Figure A1:**
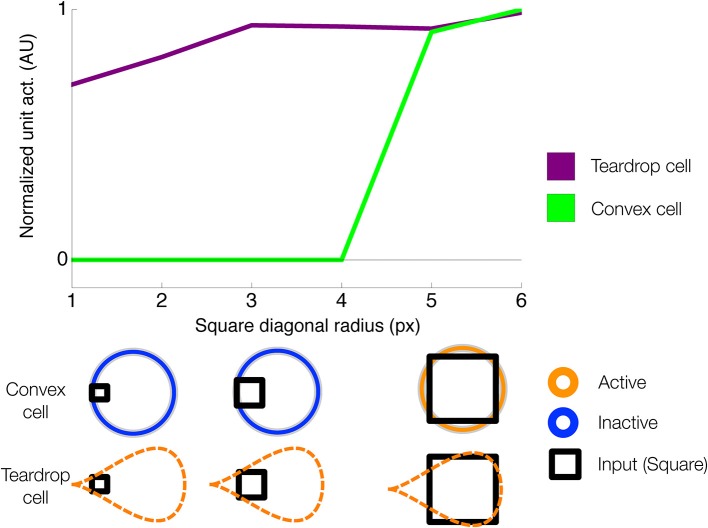
**Teardrop cells demonstrate the size invariance property**. The size of the square is labeled “1” to “6,” from smallest to largest, respectively. The activity of a convex cell (orange) and a teardrop cell (blue) is plotted when the six squares of different sizes were presented within the RF. The teardrop cell activity only changes modestly when squares of different sizes are presented (size invariance), whereas the convex cell only responds when the square reaches a certain size. The activity of each cell is normalized separately to convey differences in the response properties.

The behavior of teardrop cells is consistent with properties of cells found in area AIT of primate cortex. Teardrop cells exploit the jitter in RF size and position of neurons in the visual system. Teardrop cells have large RF sizes, by virtue of their integration of convex cell inputs from different sized RFs. Their RF size is at least as large as the largest convex cell unit that provides input. So long as the figure remains within the RF, a teardrop unit yields a response to the medial axis of a figure, irrespective of its retinal size. No attempt was made to quantitatively fit the neurophysiological properties of AIT neurons because we focused on the core figure-ground mechanisms. We selected a set of teardrop cells in our simulations with eight integration directions, corresponding to the horizontal, vertical, and diagonal directions. We found this set was sufficient to yield qualitative matches to the medial axis sensitivity of neurons. Similar to AIT neurons, teardrop cells demonstrate size invariance in their responses (Figure A1).

### Convex cell recurrent circuit

**Figure 4:**
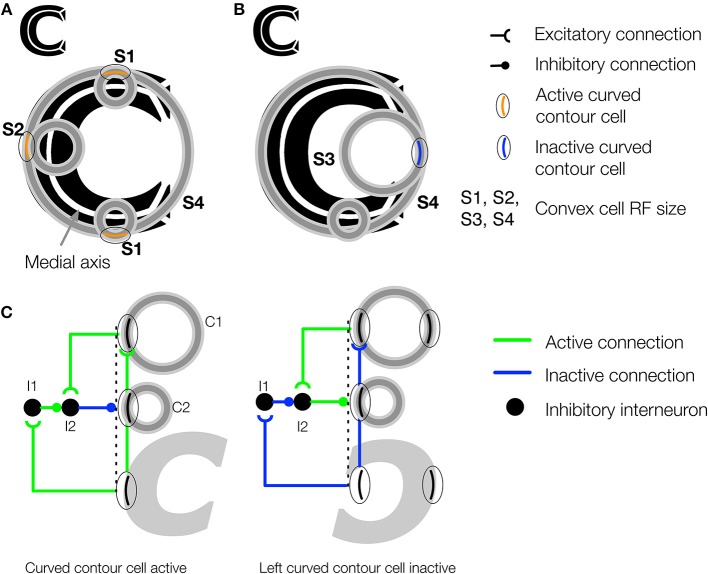
**The convex cell recurrent circuit suppresses responses outside of figures in concave regions**. **(A)** The medial axis of the C-shape is superimposed on the figure in white. Units with small RF sizes (S1 and S2) detect points along the medial axis. **(B)** Without feedback, units (S3) may incorrectly detect a medial axis within the concave region of the C-shape display. Ambiguity about the correct location of the medial axis is resolved in the model through feedback from large RF units (S4), which respond to the closure of the figure's boundary. **(C)** Proposed neural circuit for the model's convex cell recurrent feedback mechanism. Curved contour cells project to a convex cell with a large RF (C1) and to an inhibitory interneuron (I1) in the same layer as a convex cell with a smaller RF (C2). The convex cell with the large RF (C1) projects to another inhibitory interneuron (I2) that receives an inhibitory connection from I1. I2 has an inhibitory connection to the convex cell with the smaller RF (C2). When the curved contour cell and convex cell with the larger RF (C1) are both active, the inhibitory signals that act on C2 cancel out, which results an enhanced response in C2. When the curved contour cell is inactive but C1 is active, as may occur when the concavity in the C-shape appears within the RF, feedback from C1 to the interneuron I2 results in suppression of C2.

**Figure A2:**
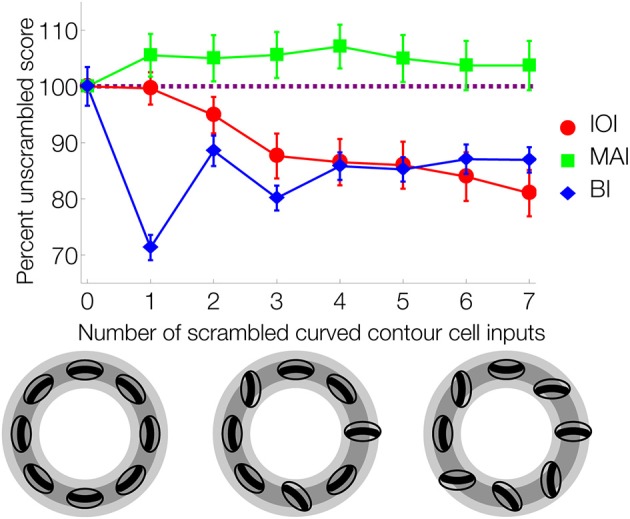
**Figure-ground and medial axis detection performance decrease when the orientation of convex cell inputs are scrambled**. The figure-ground performance (y axis) is plotted when the orientation of the indicated number (0–7) of convex cell inputs (x axis) are randomly scrambled. Performance is expressed relative to index scores garnered when convex cells group their inputs from curved contour cells in the shape of an annulus (depicted on bottom-left). Simulations were run 20 times on the 500 high complexity (HC) block displays and averaged for each number of scrambled inputs. On a given run, a number of convex cell inputs in random positions of the RF were replaced with other curved contour cells with possibly different orientations (45° increments). Error bars correspond to ±1 standard deviation. The best figure-ground and medial axis performance was achieved when convex cells had an annulus-shape RF organization. Convex cell activity was distributed closer to the boundary, away from the medial axis, when inputs were scrambled.

Although the feedforward model architecture will correctly detect the medial axis of a figure, false positive candidates may emerge in figures with concavities. Figures 4A,B shows in black a figure with a concavity called the C-shape. Its medial axis is superimposed in white. The RFs of active convex cell units with three different size RFs are shown. The set of the smallest active convex cell units that are shown (“S1” and “S2”) signals the medial axis of the C-shape to teardrop cells (Figure 4A). However, a set of convex cell units with larger RFs (“S3”) signals the presence of a false medial axis that spans the C-shape concavity, outside of the figure (Figure 4B). We propose that recurrent feedback connections between convex cell units suppress responses when they are due to a false medial axis outside the figure. The recurrent connections among convex cells are asymmetric: units only receive feedback from others with larger RF sizes. We are not aware of physiological evidence demonstrating asymmetric “coarse to fine” connectivity among cells with different RF sizes, although the idea has been used in existing theory (Grossberg, 1994). Figure 4C shows a neural circuit that implements the convex cell recurrent mechanism. An analysis of the convex cell RF organization is shown in (Figure A2).

### Teardrop cell feedback circuit

**Figure 5:**
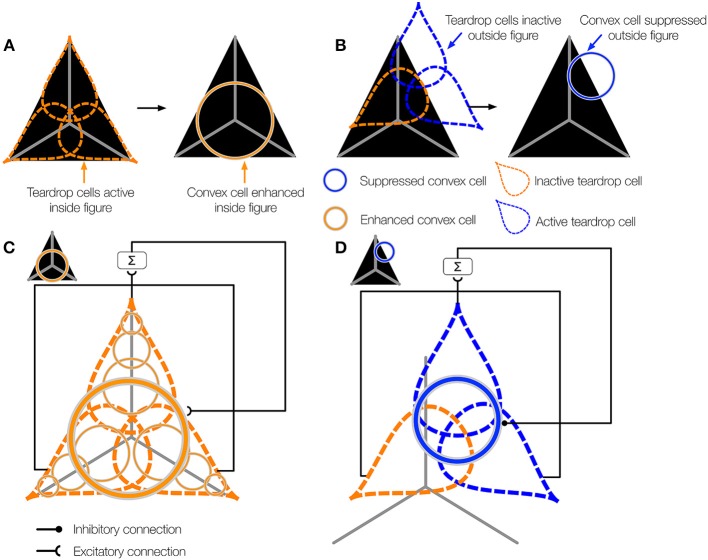
**The teardrop cell feedback circuit enhances convex cell activity along the medial axis of a figure**. **(A)** Left panel: Three teardrop cells are shown (outlined by the dashed ellipses) that group convex cells with jittered RF sizes and positions along integration directions that coincide with the medial axis of the triangle figure. Right panel: Feedback from the active teardrop cells (orange) enhances the convex cell centered on the medial axis. **(B)** Left panel: Only the bottom left teardrop cell is active because a large segment of the medial passes within its RF. The medial axis does not enter the RF of the other two teardrop cells, so they are inactive. Right panel: The convex cell shown is inhibited because two of the three teardrop cells are inactive. **(C,D)** The teardrop cell feedback circuit mechanism. **(C)** The teardrop cells in **(A)** share a common RF position, defined by the convex cell with the largest RF size from which they receive feedforward input (thick orange circle in the center). All three teardrop cells are active. Due to the good agreement in the activation of the teardrop cells that share the same RF position, feedback to convex cells in the same position that have the same sized or smaller RFs is enhanced. The feedback results in interior enhancement in convex cells with small RF sizes compared to the figure. **(D)** Only one of three teardrop cells in **(B)** is active, so it is unlikely that there is true medial axis in the RFs. Feedback from teardrop cells to the convex cells in the same position is inhibitory, which suppresses activity away from the medial axis.

When one or more teardrop cells with different integration directions that share a common RF position is active, it may be because their RFs are positioned on the figure's medial axis. Figure 5A schematically depicts the collection of teardrop cells that have different integration directions whose RFs are positioned on a triangle figure. Because each teardrop cell has the medial axis within the RF (gray line), the units are active (orange). The fact that all three teardrop cells are active provides evidence that they are positioned on the interior of a figure. If sufficiently many teardrop cells are active, they send feedback to enhance the activity of the convex cells from which they received feedforward input (Figure 5C). The feedback results in an interior enhancement signal in convex cells centered on the interior of the figure.

Consider the case when few of the teardrop cells that share the same RF position are active. In the example depicted in Figure 5B, only one teardrop cell would be active nearby the top-right corner of the triangle because a large segment of the medial axis is in the RF. The other two teardrop cells with the same RF position (blue) are inactive because they are not positioned along the medial axis of the figure. If too few teardrop cells are active, the model sends inhibitory feedback to suppress the activity of the convex cells from which they received feedforward input (Figure 5D). This prevents convex cells with RFs centered outside of the figure from demonstrating interior enhancement. In summary, the activity of convex cells on the interior of the figure is enhanced, while the activity of convex cells outside the figure is suppressed.

Convex cells represent the units in our model that demonstrate an enhanced response to the interior of a figure. Our model predicts that these cells that exhibit interior enhancement are aligned with the medial axis. We simulated convex cells with seven different RF sizes. Units with different RF sizes that share a common RF center compete in a contrast-enhancing recurrent network. Cross-scale competition sharpens the network's sensitivity to the position of the medial axis. Activity of units that do not receive input from boundary contours on either side of the RF will be suppressed.

### Visual displays

**Figure 6:**
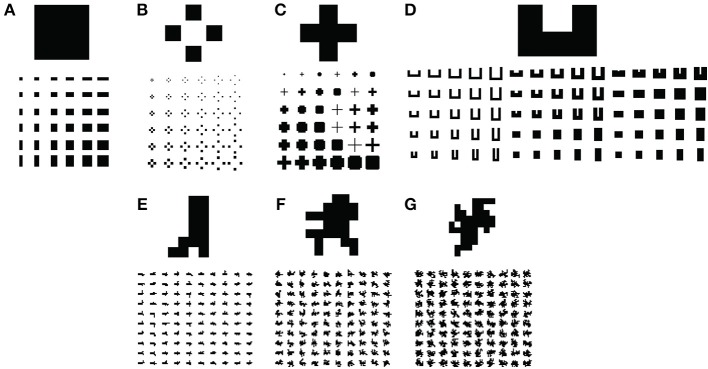
**Visual displays used in simulations to test the model**. Five hundred of each type of visual display depicted in **(E,F)** were parametrically generated. Only 100 are shown. **(A)** Rectangles. **(B)** Square textures. **(C)** Crosses. **(D)** C-shapes. **(E–G)** Algorithmically generated random block displays with low **(E)**, medium **(F)**, and high **(G)** complexity.

We sought to test the model's capabilities by simulating parametrically varying versions of figures (Figure 6) that resemble those used in electrophysiological studies of figure-ground segregation (Zipser et al., 1996; Zhou et al., 2000). We tested the model on rectangular (Figure 6A), square texture (Figure 6B), cross (Figure 6C), C-shape (Figure 6D), and randomly generated block shapes with varying complexities (Figures 6E–G). Concave regions tend to be part of the background rather than the figure and pose a challenge to models of figure-ground segregation. The C-shape and random block displays test the model's ability to avoid these regions when responding to the figure. We produced 500 low (LC), medium (MC), and high (HC) complexity random block displays, and 100 of each type are depicted in Figures 6E–G, respectively.

We parametrically varied the aspect ratio of the rectangular displays in the range 1/8 to 8, yielding 64 shapes. The aspect ratio of the C-shape was adjusted in equally spaced increments in the range 1/4 to 4 and the C-shape was 1–6 px thick to yield 96 shapes. We generated 36 crosses (6 thicknesses × 6 sizes) and square-texture displays (6 texture element displacements × 6 element sizes).

The random block displays were generated using a modified version of a random block generation algorithm (Sakai et al., 2012). The block algorithm begins with a base rectangle and iteratively adds an adjacent block to a random location along the rectangle boundary. In the iteration following the addition of a block, locations bordering either the rectangle or newly added block may be randomly selected for the next block addition. We generated LC, MC, and HC random block displays by adding 4, 16, and 32 blocks, respectively. Greater numbers of blocks afford greater complexity due to the increased irregularity in the figure boundaries. We constructed 500 unique blocks of each type in each condition.

### Figure-ground indices

To quantify model performance across the visual display sets, we define several indices that assess figure-ground responses in the model. Larger index scores indicate better performance. The In-Out-Index (IOI) provides a measure of how much convex cell activity is distributed on the interior of the figure compared to the background:

(1) $$IOI=\frac{A_{Figure}-A_{Ground}}{A_{Figure}+A_{Ground}}$$

In Equation (1), *A*_*Figure*_ and *A*_*Ground*_ refer to the mean unit activity inside the figure and ground regions, respectively.

We define two additional indices to assess the spatial distribution of model unit activation in each visual display. Equation (2) defines the medial axis index (MAI), which measures the ratio of unit activity distributed within 1 pixel of the medial axis of the figure (*A*_*Medial*_), as computed Mathematica, to the mean activity on the complementary portion of the interior of the surface (*A*_*Interior*_). Greater MAI scores indicate a greater proportion of the model activation due to the figure is distributed along the medial axis.

(2) $$MAI=\frac{A_{Medial}-A_{Interior}}{A_{Medial}+A_{Interior}}$$

Equation (3) defines the boundary index (BI), which measures the ratio between the activity distributed within 1 pixel of the boundary of the figure (*A*_*Boundary*_) and the activity garnered to the interior and exterior of the figure (*A*_*Elsewhere*_). Greater BI scores indicate that much of the model activation is concentrated around the boundary of the figure.

(3) $$BI=\frac{A_{Boundary}-A_{Elsewhere}}{A_{Boundary}+A_{Elsewhere}}$$

## Results

A central focus of the model is to better understand interior enhancement and the signaling of the medial axis of a figure by neurons in the primate visual system. We performed simulations of the model to investigate whether mechanisms in higher visual areas yield interior enhancement, which may underlie the effect observed in primary visual cortex. In Section Interior Enhancement and Medial Axis Sensitivity to Exemplar Figures, we examine medial axis detection and interior enhancement in exemplar visual displays. In Section Spatio-Temporal Dynamics, we focus on the spatio-temporal response of convex cells to show that these units do in fact exhibit interior enhancement, similar to units in primary visual cortex. In Section The Role of Feedback in Interior Enhancement and Figure-Ground Segregation, we describe performance of the model on larger numbers of visual displays, including parametrically generated figures, and analyze the role feedback has on enhanced interior responses. Appendix 3 in Supplementary Material contains the model equations.

### Interior enhancement and medial axis sensitivity to exemplar figures

**Figure 7:**
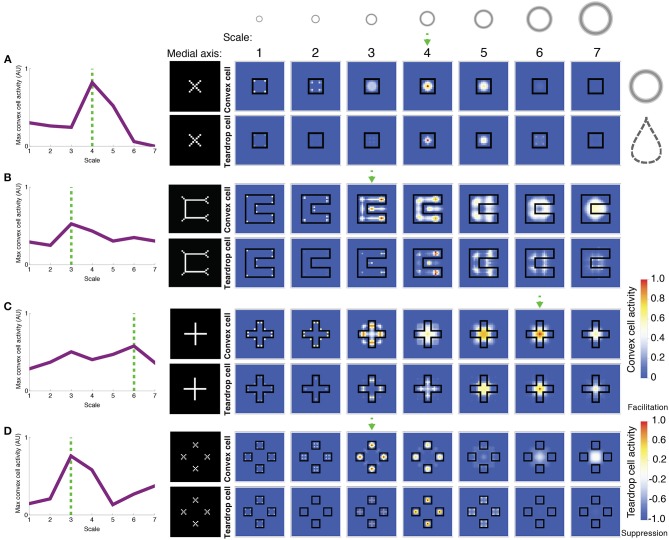
**Model simulations of exemplar figures (A–D)**. The most active convex cells (top rows of panels) signal the position of the figure's medial axis. The medial axis, as computed by Mathematica, for each figure is shown for comparison in the column to the left of the model dynamics. The degree of interior enhancement of convex cells due to feedback from teardrop cells is shown in the bottom rows of panels. Columns from left to right show the activity of small to large RF sizes, respectively, which are provided along the top row. The relative size of the RFs, compared to the visual displays, is depicted by the annuli at the top. The boundary of the simulated figures is outlined in black. The response of the most active convex cell is plotted on the leftmost column for each RF size, labeled 1–7 from small to large. The dashed green arrow and lines indicate the RF size of the most active convex cell. Note that in **(B)**, the most active convex cells have RFs centered on the medial axis of the C-shape rather than inside the concavity. While convex cells respond when their RFs are centered along *points* of the medial axis, teardrop cells collect evidence about the closure of the figure's boundary contours within the RF. Teardrop cells do this by grouping in different directions the signals from convex cells with jittered RF sizes and positions. Integrating information about the closure of the figure's boundary over an extended region affords a robust response to the interior of a figure, when the RF is positioned along the medial axis. Teardrop cells send feedback to convex cells to enhance their activity if the RF is centered on the medial axis of the figure, or suppress otherwise. Blue indicates suppression and orange/red indicates an interior enhancement signal.

To summarize the model dynamics and behavior, we often plot the activity of convex cells as a measure of the estimated location of the medial axis (e.g., Figure 7). To readout the detected location of the medial axis from the model dynamics, we consider the spatial position of the maximally active convex cell. We do not claim that the brain decodes neural signals to locate the medial axis using maximum likelihood. This approach provides a simple way to readout activity across the network.

Figure 7 depicts the activity of convex cells (top panels), which signal the medial axis, and teardrop cells (bottom panels), which signal interior enhancement. The inputs in each simulation are exemplar figures from the parametrically generated sets of visual displays shown in Figure 6. Figure 7A shows the model response to a square. Convex cells with a RF size of 4 yield the greatest activity compared to units with other RF sizes. The activity peak is concentrated at the center of the square. Convex cells with a RF size of 4 yield a MAI score of 0.91, which indicates that a high proportion of the neural activity to the figure interior is distributed along the medial axis. It is also the case that convex cells with smaller RFs yield activity peaks on the medial axis, along the diagonals of the square. The smaller the RF size, the closer the activity peaks are to the corners.

The activity of convex cells with size 5 RFs and larger is suppressed, due to inhibition from teardrop cells. Recall that the teardrop feedback circuit suppresses convex cell activity when RFs are not positioned on the medial axis of the figure. Convex cells with large RF sizes compared to the square yield broad and weak distributions of activity. The activity is not constrained to the medial axis of the square, and is therefore suppressed. The high concentration of teardrop cell activity at the center of the square in units with size 4 RFs indicates that interior enhancement occurs in convex cells whose RFs are centered on the square. Teardrop cells facilitate an augmented response in convex cells to the figure through feedback.

Figure 7B depicts the model response to a C-shape display. The C-shape represents an important test for models of figure-ground segregation because the concave region is locally similar to the C-shape interior. The greatest convex cell activity is garnered by size 3 units whose RFs are centered along the medial of the C-shape (MAI = 0.67). Therefore, the model correctly performs figure-ground segregation because the peak is located inside the C-shape rather than inside the concavity. The teardrop feedback circuit alone does not result in correct figure-ground assignment because both the interior of the C-shape and the concavity are considered in the model as candidates where a medial axis may be located. The convex cell recurrent circuit is an important component of the model that allows it to correctly identify the medial axis of the C-shape. Size 4 teardrop cells yield the maximal activity, which signals interior enhancement to convex cells.

Feedback from teardrop cells does not completely abolish activity due to the concavity, which is consistent with the recent psychophysical finding that figure-ground percepts may reverse when the shape of the concavity is manipulated (Kim and Feldman, 2009). Adjustments to the curvature or junctions of the C-shape may change whether convex cell populations inside the C-shape or the concavity are more active.

Figure 7C depicts the model response to a cross. As shown in the left panel, the distribution of the maximally activity convex cells with different RF sizes is bimodal. The peaks garnered by units with smaller and larger RF sizes correspond to a response to the medial axis along the arms and center of the cross, respectively (MAI = 0.74). Units with size 6 RFs produce a strong response to the center of the cross due to feedback signals from teardrop cells (bottom panel), yielding interior enhancement. As shown in the bottom panels, teardrop cell activity is weak outside of the cross, which results in suppression of convex cells whose RFs are centered there. There is facilitation at the interior—particularly in units whose RF sizes are comparable in the length to the arms of the cross (RF sizes 5 and 6). The secondary activity peak produced by convex cells with size 3 RFs occurs due to the convex cell recurrent circuit. Convex cells with large RFs, comparable in size to the cross send feedback signals to enhance the response of units with smaller RFs, comparable in size to the width of the arm of the cross (size 3). The enhancement in the smaller RF units occurs because the small and large RF convex cells share common inputs from curved contour cells that respond to the distal parts of the arms.

Figure 7D shows the model response to the square texture display, which tests performance when there are multiple texture elements with various sizes and displacements. The largest convex cell activity peak occurs in units with size 3 RFs (MAI = 0.83). There are four distinct activity peaks that are located at the center of each of the squares. A smaller secondary activity peak occurs in units with size 7 RFs because the RFs are sufficiently large to group the square elements across the center gap. Teardrop cells are most active at the center of the squares, which yields interior enhancement in the convex cells.

**Figure 8:**
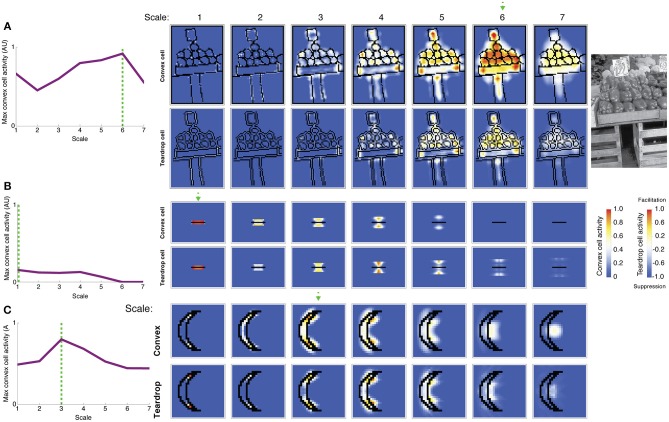
**Model simulation of images of a natural scene and a thin bar**. The most active convex cells (top rows of panels) signal the position of the figure's medial axis. The degree of interior enhancement of convex cells due to feedback from teardrop cells is shown in the bottom rows of panels. **(A)** Simulation of an image of peppers from the Berkeley Segmentation Dataset (right). Convex cells with size 6 RFs (dashed green arrow) yield the maximal response in the center of the pepper figures. **(B)** Simulation of a bar, which is thinner than the smallest convex cell RF. The most active convex cells are distributed closely to the bar, as the model does it best it can to detect the medial axis, and activity drops of precipitously with distance from the bar. **(C)** C-shape and crescent figures yield similar medial axis and interior enhancement in convex cells. The C-shape and crescent figures have comparable sizes, but the crescent boundaries are curved. The green dashed line and arrow indicate the RF size of the most active convex cell. Convex cell responses to the concave region diminished compared to the C-shape simulation (Figure 7B), indicating an improved response gain to the interior of the figure.

Figure 8A shows the activity of convex cells (top panels) and teardrop cells (bottom panels) with different RF sizes to a natural image of peppers taken from the Berkeley Segmentation Dataset. We wanted to test the model's figure-ground performance and ability to detect the medial axis in a more complex scene. The activity of convex cells with small RF sizes is distributed close to boundary contours. The peak convex cell activity occurs in units with size 5 and 6 RFs, near the center of the peppers. Teardrop cells are mostly quiescent, except for units with size 5 and 6 RFs, and clusters of activity coincide with the medial axis of the peppers. Therefore, feedback from teardrop cells facilitates an enhanced response in convex cells centered along the medial axis of the peppers. Teardrop activity diminishes in units with larger RF sizes, which indicates that the RF size is too large to integrate fine details of the scene.

In Figure 8B, we show results of a simulation of a bar that is thinner than the width of the smallest convex cell RF. We wanted to test model performance on a limiting case of when the figure has an infinitesimal width. Only convex cells with small RF sizes centered nearby the bar are active. The activity of units with larger RF sizes centered farther from the bar is greatly reduced. The spatial distribution of convex cell activity remains close to the bar and does not spread far away. The response of teardrop cells follows a similar trend: units with small RFs are active nearby the bar, and the response is lower in units with larger RFs. Convex cells with large RF sizes are not sufficiently active to overcome the suppression from teardrop feedback and are completely inhibited. This indicates that the model does the best job it can to identify a medial axis of an extremely thin figure.

We primarily tested model performance on figures with right angles, such as the C-shape and block visual displays; however, performance remained good on figures with curved contours. Consider the crescent shape shown in Figure 8C that approximates the C-shape. Convex cells whose RFs are centered along the medial axis produce the greatest response. Suppression of convex cell responses outside of the figure is greater in the crescent shape simulation, compared to the C-shape (Figure 7B). The curvature of the crescent boundary contours more closely matches the preferred sensitivity of the curved contour units than the right angles in the C-shape. This suggests interior enhancement in the C-shape would improve if model V4 included populations neurons sensitive to conjunctions of bars (Hegde and Van Essen, 2006).

### Spatio-temporal dynamics

**Figure 9:**
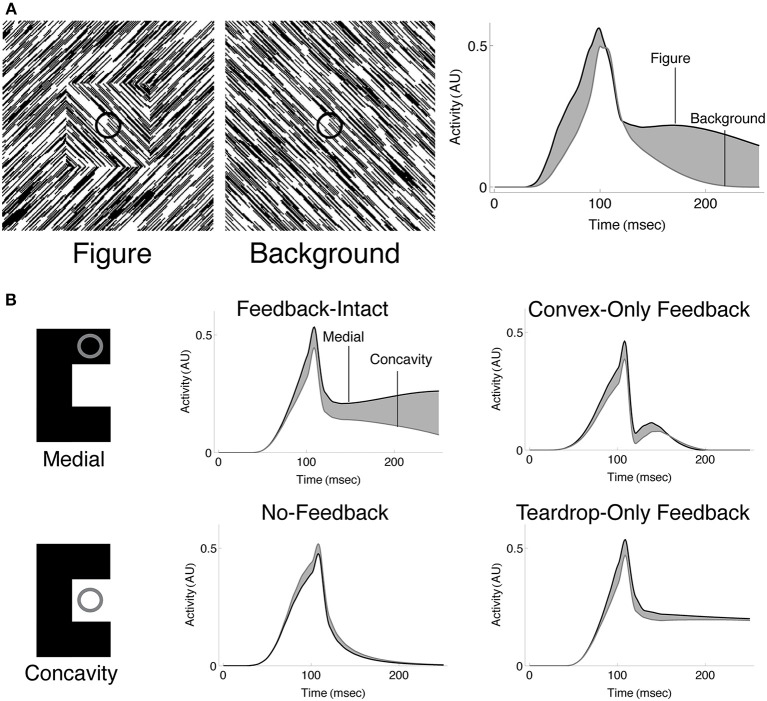
**Spatio-temporal dynamics of the model**. **(A)** Convex cells demonstrate interior enhancement similar to single cells in primate area V1 (compare with Figure 1A). We presented the model with a textured display that either contained a square figure (left panel) or just the background (middle panel). The square subtended the same size as that simulated in Figure 7A. The dynamics of the convex cell whose RF is centered on the square when it was present are shown on the right panel. The response of the convex cell is larger in the presence of the figure (black curve) than when just the background was present (gray curve). **(B)** Interior enhancement along the medial axis of a figure occurs due to feedback in the model. We presented the C-shape used in Figure 7B and plotted the dynamics of convex cells whose RFs were centered on the medial axis (*Medial*; black curves) or on the concavity (*Concavity*; gray curves). The response is enhanced to the medial axis compared to the concavity when feedback connections in the model are not lesioned. This indicates that feedback plays an important role in enhancing convex cell activity to the medial axis and suppressing activation to the background. Our model contains two types of feedback connections: the convex cell recurrent circuit and the teardrop cell feedback circuit. We considered the dynamics of the model when all feedback is intact (*Feedback-Intact* condition) and when all feedback was lesioned (*No-Feedback* condition). We also considered the effect each type of feedback connection had on model behavior through selective lesions (*Convex-Only Feedback* condition and *Teardrop-Only Feedback* condition).

We sought to investigate whether the temporal dynamics of convex cells are similar to those of neurons in V1 that show interior enhancement (Figure 1A). Following the paradigm of Lamme et al. (1999), we presented the model with a textured scene with (Figure 9A; left panel) or without (Figure 9A; center panel) a square figure. The right panel of Figure 9A shows the temporal dynamics of a convex cell with a RF size of 4 whose RF was centered on the square when it was present. Similar to the V1 neuron responses, the convex cell demonstrates an enhanced response when the figure was present. Similar to the single-cell data, most of the modulation occurs later, following the peak response. The convex cell also demonstrates some interior enhancement prior to the peak response, unlike the neural data. We suspect that this is due to the lack of conduction delays in our model. Feedback from teardrop cells arrives instantaneously, yet *in vivo* there would be a delay for the signal to propagate and act on the target population of neurons. This could shift the onset of the interior enhancement beyond the peak response.

The results shown in Figures 7, 8 suggest that feedback from teardrop cells plays an important role in enhanced responses along the medial axis. Signals from teardrop cells often suppress convex cell activity not along the medial axis, even elsewhere within the interior of the figure. We wanted to better understand the role of feedback in the temporal dynamics of interior enhancement. Figure 9B plots the temporal response of convex cells whose RFs are centered on the medial axis (top left panel) and on the concavity (bottom left panel). To investigate the importance of feedback, we selectively lesioned different feedback connections in the model. Consistent with the results shown in Figure 7B, the response along the medial axis is larger than within the concavity when feedback is intact (Figure 9B; top center panel). When feedback is completely abolished, the ordinal relationship between the concavity and medial axis responses reverses: the convex cell activity is slightly larger in the concavity than on the medial axis (Figure 9B; bottom center panel). This would indicate an incorrect figure-ground assignment by the model, and shows that feedback is responsible for enhancing activity within the C-shape interior. The dynamics in the Convex-Only and Teardrop-Only Feedback conditions are shown in the right panels. In both cases, the individual types of feedback connections yield an increased response to the medial axis relative to the concavity, but the degree of the modulation is less in either case than when both feedback connections are intact.

### The role of feedback in interior enhancement and figure-ground segregation

**Figure 10:**
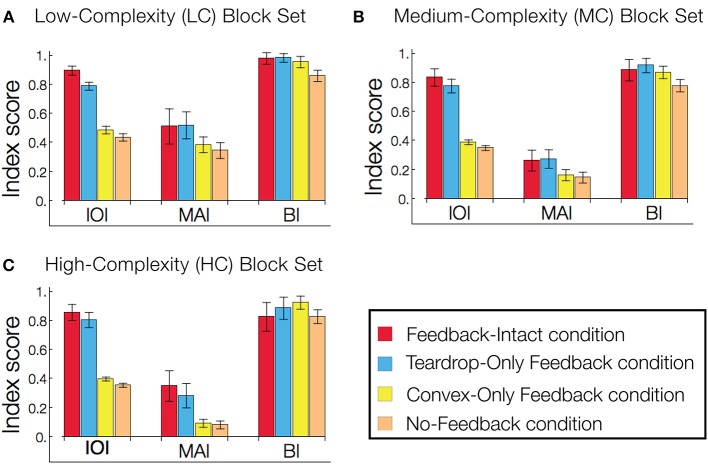
**Feedback greatly improves figure-ground segregation**. Performance is expressed with respect to the IOI, MAI, and BI. The in-out index (IOI) assesses the proportion of convex cell responses are due to the figure rather than the background. The medial axis index (MAI) measures how concentrated convex cell responses are on the medial axis of the figure. The boundary index (BI) assesses how proportions of convex cell responses occur near the boundary of the figure, compared to other regions in the display. Performance in the low-complexity **(A)**, medium-complexity **(B)**, and high-complexity **(C)** block sets is expressed with respect to the IOI, MAI, and BI. All performance indices are normalized such that 1 indicates the best performance and 0 the worst. Each bar represents the mean index score across the entire visual display set, and the error bars correspond to ±1 standard deviation. The indices are computed based on convex cell activity. Simulations are performed on the low (LC), medium (MC), and high (HC) complexity random block displays (Figures 6E–G). We assessed model performance when feedback from the convex cell recurrent circuit was lesioned (blue, *Teardrop-Only Feedback* condition), when feedback from teardrop cells was lesioned (yellow, *Convex-Only Feedback* condition), when both types of feedback were lesioned (orange, *No-Feedback* condition), and when all feedback was intact (red, *Feedback-Intact* condition). Convex cell responses were most concentrated on the interior of the figure (IOI) in the Feedback-Intact condition, and performance was the worst in the No-Feedback condition. Lesioning feedback from teardrop cells resulted in the greatest decrease in performance. For the HC display set, which contains figures with irregular boundaries and concavities, the Convex-Only Feedback condition yielded the best figure-ground performance.

The results in Figure 9 prompted us to quantify the role of feedback on interior enhancement for a broader range of visual displays. Figure 10 shows model performance as assessed by the IOI, MAI, and BI on the LC, MC, and HC random block displays. The block displays test the model's ability to detect the interior of complicated figures despite the presence of many local concavities along the irregular boundaries. The relative response to the figure compared to the background, as measured by the IOI, was greatest in Feedback-Intact condition (red), and lowest in the No-Feedback condition (green). The action of the convex cell recurrent circuit alone (Convex-Only Feedback condition, yellow) only slightly improved performance compared to the No-Feedback condition. However, the teardrop cell feedback circuit (Teardrop-Only Feedback condition) alone resulted in substantially improved selectivity to the interior of the figure compared to the background (blue). The absence of lesions (red) improved the relative response to the figure by a margin that often exceeded the combined individual gains obtained from single lesions. The Convex-Only Feedback condition scored the highest BI on the HC random block displays, which indicates a shift in the convex cell activity toward the boundary of the figure compared to the Feedback-Intact condition. The Teardrop-Only Feedback condition yielded a high MAI score, which indicates that the feedback mechanism contributes to an increased sensitivity of convex cells to the medial axis of the figure.

For the LC random block displays, the Feedback-Intact condition garnered the largest BI scores (red). However, for the HC random block displays, the Convex-Only Feedback condition (yellow) yielded the highest BI score. Given that the IOI scores for the Convex-Only Feedback condition remained roughly constant irrespective of the input complexity, the increased BI scores indicate that the convex cell recurrent feedback circuit distributed activity closer to the boundary contours, yet still within the interior of the figure.

**Figure 11:**
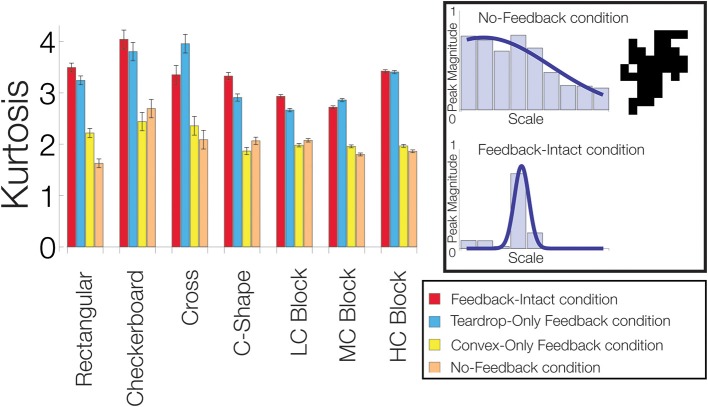
**Feedback improves the model's sensitivity to the medial axis of the figure**. For each visual display set, the kurtosis of the distribution of the most active convex cells with different RF sizes is plotted. An example of a low kurtosis distribution of most active convex cells with different RF sizes is shown on the top-right panel. The bottom-right panel shows a distribution with a high kurtosis. We compared performance when feedback from the convex cell recurrent circuit was lesioned (blue, *Teardrop-Only Feedback* condition), when feedback from teardrop cells was lesioned (yellow, *Convex-Only Feedback* condition), when both types of feedback were lesioned (orange, *No-Feedback* condition), and when all feedback was intact (red, *Feedback-Intact* condition). A high concentration of activity in units with a single RF size indicates a high degree of confidence in the medial axis response. A low kurtosis indicates that the energy in convex cell responses is more evenly distributed among units with different RF sizes. Performance was best in the Feedback-Intact and Teardrop-Only Feedback conditions.

To quantify how feedback affects the sensitivity of convex cells to the medial axis of the figure, we computed the kurtosis for the distribution of the convex cells yielding the maximal activity with different RF sizes (e.g., Figure 7, left column). Often used in statistics, the kurtosis assesses how modal or “peaked” a distribution appears. For the distribution of maximal convex cell activity, the measure provides a diagnostic to assess how effectively feedback enhances the units with an appropriate RF size to code the medial axis. A large kurtosis indicates that most of convex cells that are active have a common RF size (Figure 11, lower-right panel). A high concentration of activity in units with a single RF size indicates a high degree of confidence in the medial axis response. A low kurtosis indicates that the energy in convex cell responses is more evenly distributed among units with different RF sizes (Figure 11, top-right panel). A broad distribution indicates a lack of confidence in the medial axis response.

In the majority of the visual display sets we tested (6/7), the Feedback-Intact condition yielded the greatest kurtosis, which suggests that feedback increases the confidence and selectivity of convex cell responses to the medial axis of the figure. The Teardrop-Only Feedback condition generally yielded the next greatest kurtosis. The Convex-Only Feedback condition alone often did not yield a much larger kurtosis than in the No-Feedback condition. This indicates the convex cell recurrent circuit, as presently configured, did not increase the concentration of activity along the medial axis. From Figure 10, as the complexity of the block sets increased, the MAI decreased while the BI increased in the Convex-Only Feedback condition. Given the low kurtosis values, this suggests that convex cell feedback disperses activity more evenly within the figure surface.

In summary, the considerably greater kurtosis in the Feedback-Intact and Teardrop-Only Feedback condition conditions compared to the No-Feedback condition suggest that feedback plays a crucial role in increasing the response gain to the medial axis of a figure. Feedback also increased the confidence of model responses about the location of the medial axis.

## Discussion

We presented the teardrop model of figure-ground segregation in the primate ventral stream that explains why neurons demonstrate enhanced activity when their RFs are centered on the interior of a figure compared to the background. Our results support the possibility that interior enhancement arises as the result of dynamical interactions between higher visual areas. The proposed model makes the major theoretical prediction that interior enhancement originates in convex cells and the effect propagates via feedback to cells in earlier visual areas (Lamme, 1995; Lee et al., 1998). More specifically, we predict that cells in area PIT demonstrate interior enhancement prior to those in V1 due to recurrent interactions and feedback from teardrop cells. We also predict that teardrop cells that exploit jitter in convex cell RFs play an important role in modulating the interior enhancement effect. Our model is based on the following three propositions.

Proposition 1: Neurons that demonstrate an enhanced response to the interior of a figure signal the presence of the medial axis. Indeed, the responses of IT neurons support a representation of figures using its medial axis representation (Hung et al., 2012). The “late component” response of neuron in ventral areas not only is associated with interior enhancement, but also to the medial axis of shapes (Hung et al., 2012). Neurons in V1 that demonstrate interior enhancement show elevated responses at the center of texture-defined figures during the “late component” stage, which is consistent with medial axis coding (Lee et al., 1998). Our simulation results show that model convex cells demonstrate enhanced activity along the medial axis of figures due to dynamical cooperative/competitive interactions between higher visual areas. We propose that feedback signals from convex cells to earlier visual areas may form the basis of the interior enhancement effect observed in V1 neurons. The mechanisms in the present model explain how responses in small RF units are constrained to the medial axis of a figure, which affords a parsimonious and efficient representation of a figure.

Proposition 2: There is a purpose for neurons with different RF sizes in areas in the visual system, aside from potentially detecting and representing figures of different sizes. The existence of neurons with multiple RF sizes in areas throughout the visual system is well known, yet their role is not clear. We claim that jitter in RF size and position serves a crucial role in figure-ground segregation. In our model, teardrop cells demonstrate the advantages that the visual system may garner by exploiting jitter. Grouping of signals from units with different RF sizes and positions by teardrop cells not only leads to a robust detection of a figure's medial axis, but it affords sensitivity to the closure of the figure's boundary contours. The closure of a figure's boundary contours facilitates its detection and the visual system more rapidly detects closed rather than open figures (Elder and Zucker, 1993; Mathes and Fahle, 2007; Wagemans et al., 2012). Sensitivity to closure underlies how the model successfully performs figure-ground segregation in the case of partially concave figures, such as the C-shape.

Proposition 3: Feedback plays a crucial role in yielding enhanced responses to the interior of figures. Our simulations show how interior enhancement occurs in convex cells due to feedback signals from teardrop cells. When we lesioned feedback connections in the model, convex cell activity was less concentrated along the medial axis, and across the population, there was more “false positive” activation outside the interior of the figure. The action of the teardrop feedback circuit in the model is consistent with existing models (Supèr and Romeo, 2011) and single-cell data (Supèr and Lamme, 2007) that indicate that feedback enhances the response to the figure and suppresses responses to the background.

### Teardrop cell RFs

In the model teardrop cells group signals from convex cells with jittered RF sizes and positions. In simulations, we assume for simplicity that teardrop cells integrate the signals from convex cells in equally spaced positions along each integration direction. The integration directions extended equally in all radial directions (i.e., isotropic). It is unclear how the visual system would perform the grouping, but that neurons analogous to teardrop cells likely group signals in irregular directions with variable spacing. A consequence of only considering isotropic teardrop cells is that they yield optimal responses to shapes with certain aspect ratios. The response of a teardrop cell to a square (Figure 7A) is more concentrated at the center of a square than it would be to an elongated rectangle. We found that varying the aspect ratio of figures did not qualitatively impact figure-ground segregation performance, but it yielded broader, less punctate teardrop activation along the medial axis. Note that this is simply an artifact of making simplifying assumptions for the purposes of simulation. The variability of RF configurations in cortex would be expected to yield comparable responses to figures, irrespective of the aspect ratio. Cortical magnification likely impacts the distribution of RF sizes of convex cells grouped by teardrop cells. An extension of the present model could investigate how these factors impact interior enhancement signals.

### Representation of concavities

When interpreting the model results for the C-shape, we assume that the concavity is part of the background rather than the foreground. That is, boundaries separating the C-shape and the concavity are grouped with the C-shape rather than the concavity. However, Kim and Feldman found that manipulations to the salience and shape of the concave region might locally reverse border-ownership along different parts of the C-shape boundary, which is inconsistent a globally concave percept of the “negative part” (Kim and Feldman, 2009). Consistent with the possibility that local border-ownership signals may differ from the global interpretation, a substantial number of model convex cells with small RF sizes were active within the “negative part.” “Votes” for the presence of a medial axis from the population of convex cells whose RF is centered on the interior of the C-Shape are at odds with those from the competing population whose RFs are centered on the concavity. In the model, the convex cell recurrent circuit enhanced the response of convex cells whose RFs are centered on the C-shape medial axis and suppressed those centered on the concavity. Perhaps the local reversals in border-ownership stem from reversals in the winning populations of convex cells with small RFs. The size and shape of the C-shape “negative part” may modulate the strength of the convex cell recurrent circuit and impact the likelihood that one of the populations win out.

### Medial axis coding

Populations of neurons in IT maintain a selective response when 3D rotations of the same figure are presented, which has led to the hypothesis that IT neurons may code shape with respect to a 3D interpretation rather than a set of 2D image features (Janssen et al., 2000; Yamane et al., 2008; Hung et al., 2012). Tuning to the medial axis in IT may similarly occur in 3D (Hung et al., 2012), though presently available evidence is limited. While 3D shape and medial axis tuning makes ecological sense, present data also support coding of 2D figures. IT neurons are well known to exhibit selectivity to line drawing displays and 2D projections of 3D shapes (Logothetis et al., 1995), as well as invariance to planar transformations of planar figures (Ito et al., 1995). Together, these data support the joint coding of 2D and 3D shape in IT cortex, though the primacy of one representation over the other is unclear. For example, Yamane and colleagues found robust tuning in IT neurons to shapes over a range of low-level image manipulations, such as shading, but tuning specificity declined when depth cues were removed (Yamane et al., 2008). On the other hand, Kovacs and colleagues found consistent tuning to 2D caricatures of 3D shapes (Kovács et al., 2003). The mechanisms in our model are agnostic to the issue of 2D vs. 3D coding in IT. The aim of the present paper was to test the core mechanisms of the model, so we focused on simple 2D figures. Modules for binocular disparity, shading, and other depth cues may be integrated into the model to test for 3D selectivity. However, this is outside the scope of the present paper.

### Model limitations

The manner in which teardrop cells combine their inputs likely differs from that of neurons in cortex that respond to the medial axis of figures. In particular, the distribution of RF sizes in each integration direction is unknown, and additional physiological work is required to determine whether regularity exists in how neurons in integrate their inputs. Cortical magnification and eccentricity further complicate the picture. An on-surround RF organization also likely represents a significant simplification of the great diversity of RF shapes in cortex.

We did not directly model inhibitory feedforward inputs, although recurrent competition and feedback may afford functionally similar behavior. Others have proposed that inhibitory RF surrounds emerge through feedback, rather than feedforward processes (Hupé et al., 1998). Our model only employs units with convex on-surround RF organizations. Yet, V4 and PIT are functionally diverse areas (Brincat and Connor, 2006; Hegde and Van Essen, 2006), and neurons analogous to teardrop cells in cortex may group their inputs in both convex and concave configurations, which could increase the specificity of figure-ground responses. Our simulations demonstrate that the on-surround RFs are sufficient to detect the medial axis and obtain enhanced responses to the interior of figures.

### Comparison with other models

Our model is not the first to propose that the medial axis of a figure provides a means for the visual system to represent surfaces. The medial axis serves as an attractor in the Bayesian model of Froyen and colleagues for border-ownership signals such that they are directed toward the interior of a figure (Froyen et al., 2010). Pizer and colleagues developed the Core theory in which a figure is decomposed by the visual system in terms of the boundary, width, and medial axis (Pizer et al., 1998). Unlike existing models, ours is the first to propose that interior enhancement represents the mechanism by which the visual system codes a figural surface with respect to its medial axis.

Another crucial difference is that our model presupposes the recruitment of units in higher visual areas (e.g., PIT, AIT) to determine the medial axis of a figure. A recent computational study has proposed that the visual system only requires areas V1 and V2 to determine the medial axis of a figure (Hatori and Sakai, 2014). Border-ownership signals are first determined among units in model V2, and then the medial axis is resolved through synchronous feedback to area V1. That is, the medial axis computation depends on border-ownership signals, unlike in our model. While populations of neurons involved in coding border-ownership may interact with those in our model in higher visual areas, we demonstrated that the medial axis computation need not depend on border-ownership. An elevated response occurs along the medial axis in the model of Hatori and Sakai (2014) because feedback signals from border-ownership units at boundary contours to either side arrive simultaneously and constructively interfere. It is unclear how the border-ownership signals would be synchronized across cortex, though oscillation is one potential mechanism. However, the existence of coherent oscillations in the context of image feature representations has not been proven and remains controversial (for a discussion see Craft et al., 2007). Moreover, the model of Hatori and Sakai (2014) uses units with a single spatial scale, so it unclear how the model would determine the medial axis for figures much larger than the RF sizes of V1 and V2 units. By contrast, cooperative and competitive dynamics between units with multiple jittered RF sizes are fundamental in our mdoel for estimating the medial axis. Because units with multiple RFs are at the crux of our model, the model results are robust to figures that have a range of sizes (see Appendix 1 in Supplementary Material).

Whereas the balancing of feedforward and feedback signals is critical in the present model, other models have exclusively used feedforward connections. Supèr and colleagues have presented a spiking three level network that uses a combination of excitatory inputs and surround inhibition between model layer connections to determine border-ownership and perform figure-ground segregation (Supèr et al., 2010). The model of Sakai and Nishimura also performs border-ownership assignment using asymmetric feedforward surround modulation: units signal a preferred side-of-figure response when the figure falls within facilitatory rather than the inhibitory subfield of the RF (Sakai and Nishimura, 2006). While surround modulation likely plays a crucial role in figure-ground segregation (Walker et al., 1999), we believe feedforward processing alone is too rapid to account for the delayed interior enhancement latency. The effect does not occur in primary visual cortex until ~80–100 ms following the onset of the figure (Lee et al., 1998), yet surround modulation only requires ~7 ms, an order of magnitude faster (Knierim and Van Essen, 1992). In early cortical areas, feedforward signals propagate at ~2.24 m/s (Girard et al., 2001). For example, this means that a feedforward signal only requires ~9 ms to travel from V2 to V4. Recurrent processing and feedback loops with higher visual areas require additional time and may account for the difference in the latency.

## Conclusions

The enhanced response to the interior of a figure by neurons in primary visual cortex may provide insight into how the visual system performs figure-ground segregation. We presented a model that tests the possibility that interior enhancement arises through dynamical feedforward and feedback interactions between higher visual areas. Our results support the idea that interior enhancement arises in higher visual areas along the medial axis of a figure, and the resulting signals may modulate the activity of neurons in primary visual cortex through feedback. We showed that jitter in RF size and position provides an efficient means for the visual system to determine the medial axis of a figure.

### Conflict of interest statement

The authors declare that the research was conducted in the absence of any commercial or financial relationships that could be construed as a potential conflict of interest.
