# Supplementary material for: Neural dynamics of feedforward and feedback processing in figure-ground segregation
Source: Front Psychol. 2014 Sep 10;5:972. doi: 10.3389/fpsyg.2014.00972 (PMC4193330; doi:10.3389/fpsyg.2014.00972)
Supplement: Supplementary file 1 [file DataSheet1.DOCX]

1. **Appendix 1: Teardrop cells demonstrate size invariance**

**Figure A1** shows the results of a simulation that demonstrates the size-invariance property in teardrop cells, similar to neurons in AIT (Vogels & Orban, 1996). We examined how the activity of a single convex cell and teardrop cell changed when squares of different sizes appeared within their RFs. Variations in the size of square only had a modest impact on the teardrop cell activation. That is, the activity garnered to the smallest square did not differ much from that garnered to the largest square. The dynamic range of teardrop cell responses is limited, and the response readily saturates. By contrast, the convex cell exhibited an enhanced response to the square when its size was comparable with that of the RF. The unit did not respond to the four smallest squares. The convex and teardrop cell activity curves are normalized separately to illustrate differences in the pattern of activation as a function of figure size rather than any differences in the relative magnitudes.

1. **Appendix 2: The shape of the convex cell receptive field**

In the model, we assume curved contour representations are grouped in an on-surround configuration. On-surround, annular-shaped RFs are ideally suited to detect the medial axis of a figure and are consistent with known physiology of the primate ventral stream (Hegde & Van Essen, 2006; Pollen et al., 2002; Yau et al., 2012). However, the visual system likely combines curved contour representations in multiple ways, many of which likely do not map onto an ‘ideal’ annular shape. We ignored the variability in RF organization in order to focus on the core computational mechanisms. We wanted to investigate the consequence of using minimal annular RF arrangements had on the model’s ability to detect the medial axis and perform figure-ground segregation. To test this, we ‘scrambled’ the orientation of some number of curved contour units that provide feedforward input to convex cells. The more curved contour units whose RF orientation are scrambled, the less ‘annular’ the convex cell RF appears.

**Figure A2** shows model performance according to the IOI, MAI, and BI when the orientation of curved contour unit inputs to convex cells orientations is scrambled. The IOI decreased monotonically as larger numbers of curved contour units were scrambled, which indicates a decreased response to the figure compared to the background. In other words, enhanced responses elicited by convex cells to the interior of the figure diminished. The IOI and MAI decreased as the BI increased. This suggests that non-annular RF configurations distributed activity more uniformly throughout the interior of the shape, with an increased concentrated along the boundary. Convex cell activity was less constrained to the medial axis, compared to when the RF was annular. These results suggest that annular RF configurations may serve a particularly important role in interior enhancement and yielding sensitivity to the medial axis of a figure.

1. **Appendix 3: Model Equations**

The dynamics of cells in the model are controlled by shunting first-order differential equations (Grossberg, 1973). The activity of cells ranges from 0 to 1. We use bold notation in model equations to indicate that operations apply model cells positioned at all spatial locations. Parameter values remained fixed throughout all simulations, and are listed in the text.

The following equation describes the dynamics of V4 curved contour cells $\boldsymbol{C}_{\theta}^{s}$.

|  | ${\frac{d}{dt}\boldsymbol{C}}_{\theta}^{s}=-\alpha\boldsymbol{C}_{\theta}^{s}+(1-\boldsymbol{C}_{\theta}^{s}) \boldsymbol{Q}_{\theta}^{s}\times\boldsymbol{A}$ |  |
| --- | --- | --- |

In Eq. 4, $\boldsymbol{A}$ signifies the input from the preprocessed edge map of each visual display, $\boldsymbol{Q}_{\theta}^{s}$ is the curved contour kernel, the $\times$ operator defines component-wise multiplication, and $\alpha$ is the passive decay rate of the cell to equilibrium in the absence of input. In all equations, we set $\alpha=0.5$. To construct curved contour kernels, we first generate an annulus. The following equation takes $(s,\theta)\to(x_{a},y_{a},z_{a})$, where $s$ is the radius of the annulus, $\theta\in[0, 2\pi)$, and ${(x}_{a},y_{a},z_{a})$ indicate the Cartesian coordinates of the annulus.

|  | $\left( x_{a},y_{a},z_{a} \right)=\left( s\times\sin\left( \theta\right),s\times\cos\left( \theta\right),\left( \frac{1}{2}1-\cos\left( \frac{2\pi}{s} \right) \right) \right)$ |  |
| --- | --- | --- |

We extract the curved contour kernels $\boldsymbol{Q}_{\theta}^{s}$ from eight equally sized sectors of the annular kernel:

|  | $\boldsymbol{Q}_{\theta}^{s}=\left\{ \left( x_{a},y_{a},z_{a} \right) \vert\theta_{i}\in\left\{ -\pi,-\frac{3\pi}{4},\cdots,\frac{3\pi}{4} \right\},\theta_{i-1}\leq{tan}^{-1}\left( \frac{y_{a}}{x_{a}} \right)\leq\theta_{i} \right\}$ |  |
| --- | --- | --- |

The dynamics of PIT convex cells $\boldsymbol{S}^{s}$ obey the following shunting equation. Convex cells compete with one another across scale in a contrast-enhancing network.

|  | ${\frac{d}{dt}\boldsymbol{S}}^{s}=-\alpha\boldsymbol{S}^{s}+\left( 1-\boldsymbol{S}^{s} \right)\left( \left( \boldsymbol{S}^{s} \right)^{2}+\boldsymbol{I}^{s} \right)-\left( \boldsymbol{S}^{s}+\gamma\right)\left( \sum_{k\neq s} \left( \boldsymbol{S}^{k} \right)^{2}+\boldsymbol{R}^{s}+\boldsymbol{T}^{s} \right)$ |  |
| --- | --- | --- |

In Eq. 7, $s\in\left\{ 1,\cdots,\hat{s} \right\}$ indicates the RF radius of the convex cell in pixels, where $\hat{s}$ is the largest RF radius, and $\gamma$ indicates the hyperpolarizing lower boundary of the cells, which we fixed to 0.4. In model simulations, we set $\hat{s}=7.$ $\boldsymbol{I}^{s}$ defines the feedforward input signal convex cells receive from curved contour cells. The feedforward input signal to convex cells ($\boldsymbol{I}^{s}$ ) increases nonlinearly as a function of the number of curved contour cells that are active in the RF. A greater the number of active curved contour cells yields a greater input signal.

|  | $\boldsymbol{I}^{s}=\rho(s)\times log\left( 1+\sum_{i=1}^{n} \boldsymbol{C}_{\theta_{i}}^{s}+\sum_{i=1}^{n} \sum_{j>i}^{n} \boldsymbol{C}_{\theta_{i}}^{s}\boldsymbol{\times C}_{\theta_{j}}^{s}+\sum_{i=1}^{n} \sum_{j>i}^{n} \sum_{k>j}^{n} \boldsymbol{C}_{\theta_{i}}^{s}\boldsymbol{\times C}_{\theta_{j}}^{s}\boldsymbol{\times C}_{\theta_{k}}^{s}+\cdots\right)$ |  |
| --- | --- | --- |

The reason for the increasing number of product terms in Eq. 8 (up to three shown prior to the …), is to detect co-occurrences between some number of curved contour cells $\boldsymbol{C}_{\theta}^{s}$ with different orientations, $b$, which may range up to 8 in our simulations. The terms in each sum, indexed by $i,j,k,\ldots$ correspond to all possible permutations of size $b$ of curved contour cells. The multiplication yields increases in the input signal if curved contour units at different positions in the annulus are co-active, which enhances responses to corners, junctions, and closed regions. The result is passed through a logarithmic nonlinearity to compress the range of the signal, and one is added to make the signal nonnegative. The function $\rho\left( s \right)=1+5s$ gives input signals at larger scales greater weight.

In Eq. 7, $\boldsymbol{R}^{s}$ indicates the feedback signal each convex cell $\boldsymbol{S}^{s}$ receives from other convex cells at different scales (convex cell recurrent circuit). Each convex cell $\boldsymbol{S}^{s}$ receives feedback from convex cells that have a larger RF size and share a common input from curved contour cells (see Figure 4c). Note that the RF centers are at different visuotopic positions.

|  | $\boldsymbol{R}^{s}=\frac{1}{n\times(\hat{s}-s)}\sum_{r=1}^{n} \sum_{k>s}^{\hat{s}} f\left( \boldsymbol{C}_{\theta_{r}}^{s},\kappa_{PIT},\Gamma_{PIT} \right)\times\boldsymbol{K}_{r}^{k}*\boldsymbol{S}^{k}$ |  |
| --- | --- | --- |
|  | $f\left( w;\kappa,\Gamma\right)=\tanh\left( \kappa\times w-\Gamma\right)$ |  |

In Eq. 9, the * operator indicates convolution and $\boldsymbol{K}_{r}^{k}$ is a binary indicator kernel that picks out convex cells $\boldsymbol{S}^{k}$with larger RFs ($k>s$) that have spatially overlapping curved contour cell inputs. The function $f\left( \cdot,;\kappa,\Gamma\right)$ defined by Eq. 10 is a sigmoid that ranges from -1 to 1 and acts on the curved contour cell inputs that each convex cell $\boldsymbol{S}^{s}$ has in common with those with larger RFs $\boldsymbol{S}^{k}$. If the $r^{th}$curved contour cell is inactive, $f\left( \boldsymbol{C}_{\theta_{r}}^{s} \right)$ yields a negative value, which contributes a suppressive input to the sum. Conversely, an active curved contour cell yields a positive value, which contributes an enhancing input to the sum. When $\boldsymbol{R}^{s}>0$, $\boldsymbol{S}^{s}$ is enhanced by the convex cell recurrent circuit (Figure 4c, left panel). When $\boldsymbol{R}^{s}<0$, $\boldsymbol{S}^{s}$ is suppressed (Figure 4c, right panel). The function $f\left( \cdot,;\kappa,\Gamma\right)$ implements the behavior of the interneurons depicted in Figure 4c.

In Eq. 10, $\kappa_{PIT}$ adjusts the steepness of the sigmoid’s slope and $\Gamma_{PIT}$ shifts the sigmoid horizontally. We set $\kappa_{PIT}=5$ and $\Gamma_{PIT}=1.5$.

In Eq. 7, $\boldsymbol{T}^{s}$ defines the feedback signal each convex cell $\boldsymbol{S}^{s}$ receives from teardrop cells (teardrop cell feedback circuit). Each convex cell receives feedback from the teardrop cells $\boldsymbol{M}_{d}^{s}$ with $m$ integration directions, where $d$ indexes the integration direction (Figure 5). In our simulations, we used $m=8$ integration directions. The position of a teardrop cell’s RF is determined by the largest feedforward input it receives from convex cells. Convex cells with RF size $s$ receive feedback from teardrop cells that receive their largest feedforward input from convex cells with RF size $s.$

|  | $\boldsymbol{T}^{s}=f\left( \sum_{k=1}^{m} \boldsymbol{M}_{k}^{s} {;\kappa}_{AIT},\Gamma_{AIT} \right)$ |  |
| --- | --- | --- |

In Eq. 11, the activity of teardrop cells with different integration directions at a single visuotopic position is summed and passed through a sigmoid (Eq. 10), with $\kappa_{AIT}=1$ and $\Gamma_{AIT}=0.7$.

The dynamics of AIT teardrop cells $\boldsymbol{M}_{d}^{s}$ obey the following shunting equation. Teardrop cells compete with one another across integration direction.

|  | $\frac{d}{dt}\boldsymbol{M}_{d}^{s} =-\alpha\boldsymbol{M}_{d}^{s} +\left( 1-\boldsymbol{M}_{d}^{s} \right)\left( g\left( \boldsymbol{M}_{d}^{s} \right)+\boldsymbol{J}_{d}^{s} \right)-\boldsymbol{M}_{d}^{s} \left( \sum_{k\neq d} g\left( \boldsymbol{M}_{k}^{s} \right) \right)$ |  |
| --- | --- | --- |
|  | $g\left( w;\xi,\Gamma\right)=\frac{\left( \left[ w-\Gamma\right]^{+} \right)^{2}}{\xi+\left( \left[ w-\Gamma\right]^{+} \right)^{2}}$ |  |

In Eq. 12, $g\left( \cdot,;\xi,\Gamma\right)$ defines a sigmoidal signal function (Eq. 13) that controls the behavior of the recurrent feedback across the network (Grossberg, 1973). The function induces winner-take-all, pattern preserving, or uniformizing behavior, depending on the network activity level $w$. The parameters $\xi$ and $\Gamma$ adjust the threshold and slope of the sigmoid, respectively. We set $\xi=0.05$ and $\Gamma=0.4.$ $\boldsymbol{J}_{d}^{s}$ describes the input signal teardrop cells receive from convex cells.

|  | $\boldsymbol{J}_{d}^{s}=\upsilon(s)\times log\left( 1+\frac{1}{B_{1}}\sum_{u} \left( \boldsymbol{K}_{d}^{u}*\boldsymbol{S}^{u} \right)+\frac{1}{B_{2}}\sum_{u} \sum_{v} \left( \boldsymbol{K}_{d}^{u}*\boldsymbol{S}^{u} \right)\times\left( \boldsymbol{K}_{d}^{v}*\boldsymbol{S}^{v} \right)+\frac{1}{B_{3}}\sum_{u} \sum_{v} \sum_{w} \left( \boldsymbol{K}_{d}^{u}*\boldsymbol{S}^{u} \right)\times\left( \boldsymbol{K}_{d}^{v}*\boldsymbol{S}^{v} \right)\times\left( \boldsymbol{K}_{d}^{w}*\boldsymbol{S}^{w} \right)+\cdots\right)$ |  |
| --- | --- | --- |

Eq. 14 resembles Eq. 8, the input signal to convex cells. In Eq. 14, there are $s$ inner summations, equal in number to the scale of the largest convex cell input the teardrop cell. The $i^{th}$ inner summation is normalized by $B_{i}$, the $i^{th}$ Bell number, which specifies the number of ways to partition a set of size $i$ into $q$ nonempty subsets. $\boldsymbol{K}_{d}^{j}$ is a binary indicator kernel that picks out the convex cell $j$ pixels away in the integration direction $d$. The variables $u, v, w,\ldots$ index scales of combinations of convex cells in direction $d$, where $u, v, w,\ldots\leq s$. The result is passed through a logarithmic compressive nonlinearity and the function $\upsilon\left( s \right)=log(s)$ gives greater weight to teardrop cells with larger RF sizes.

Simulations of the model were performed in Wolfram Mathematica 9 on a 2.66 GHz Apple Mac Pro computer with 64 GB of memory. Numerical integration of the model dynamics was implemented in C++ using Euler’s method with a step size of 0.1 msec.
